# An epidemiological study of a patient population, triage category allocations and principal diagnosis within the emergency centres of a private healthcare group in the Emirate of Dubai, United Arab Emirates

**DOI:** 10.1002/nop2.518

**Published:** 2020-05-26

**Authors:** Enrico Dippenaar

**Affiliations:** ^1^ Division of Emergency Medicine University of Cape Town Cape Town South Africa; ^2^ Emergency Medicine Research Group Anglia Ruskin University Chelmsford UK

**Keywords:** patient population, private health care, Triage, United Arab Emirates

## Abstract

**Aim:**

To describe, compare and correlate the number of patients seen, their demographics, triage category allocations and principal diagnosis in four emergency centres; to better understand the patient population and triage practices in this setting.

**Design:**

An observational, cross‐sectional, epidemiological study.

**Methods:**

Electronic medical records were retrospectively evaluated from patients triaged in each of the four emergency centres over six months. Descriptive statistics were used to describe the patient demographics and variance between triage category allocations.

**Results:**

A total of 56,984 patient records were captured, with an equal gender split and the workforce being the largest patient population (20–50 years). Acute upper respiratory infection was the most prolific diagnosis, and lower acuity triage categories were allocated the most. There were inconsistencies in the application of triage systems between the emergency centres, the most obvious being the variance in triage system selection and application.

## 1 INTRODUCTION

Epidemiological studies are important to determine the health needs of a population and to drive positive change where required. No such studies have been conducted in the private healthcare sector in the United Arab Emirates, with records only coming from governmental health authority reports. Summary tables such as those from the National Hospital Ambulatory Medical Care Survey, presented by the Centres for Disease Control and Prevention in the United States, show that key distributions could affect policy and regulatory changes for improved health care (Rui, Kang and Ashman, 2016).

## 2 BACKGROUND

Triage is a process whereby a patient's acuity is identified and classified from their injury or illness, and then prioritized to receive appropriate and timely medical care (Vatnøy, Fossum, Smith, & Slettebø, 2013). The application of defined triage systems has become an international norm in emergency centres. This norm, however, varies greatly across the world, with a dozen recognized triage systems developed across the globe in the last thirty years. Stemming from a basic sorting of military casualties, triage systems have become more complex in the modern healthcare setting (Kennedy, Aghababian, Gans, & Lewis, 1996). The complexity of these systems does not necessarily translate to difficult application, but rather refers to the depth of clinical evidence used in their design and development (Moll, 2010). The design of a triage system and which is the “best” remains a topic of debate. The USA, Canada, UK and Australia have been the front‐runners in the development of formalized emergency centre triage systems, with South Africa, Sweden and some Asian countries having developed their own unique systems in recent times (Forsman, Forsgren, & Carlström, 2012). With the variety of triage systems available, it can be concluded that there is no single “best‐fit” system, as triage systems are subject to differing population dynamics, disease profiles and available resources.

The largest private healthcare group in the United Arab Emirates have been using four international triage systems in their four emergency centres in the Emirate of Dubai at the time of this study (Mediclinic Middle East, 2014). These triage systems included the following: the Canadian Triage and Acuity Scale (CTAS), the Manchester Triage System (MTS), the Emergency Severity Index (ESI) and the South African Triage Scale (SATS). The deployment of triage systems across their emergency centres has been a matter of circumstance as no nationally prescribed system exists in the UAE (Fares et al., 2014). With the large number of expatriate private healthcare professionals came the influence of triage systems brought into service from their home countries (Dubai Health Authority, 2013).

The aim of this study was to describe, compare and correlate the number of patients seen, their demographics, triage category allocations and principal diagnosis in the four emergency centres; to better understand the patient population and triage practices in this setting. This study formed part of a larger research project that aimed to design and develop a standardized locally appropriate triage system based on the needs of their patient population (Dippenaar, 2016). The triage systems used and their associated patient flow timeframes have been described elsewhere (Dippenaar, 2019).

## 3 DESIGN

An observational, cross‐sectional, epidemiological study was conducted through the retrospective evaluation of patient medical records from the four emergency centres (two Urban near the city centre and two Suburban south of the emirate) of the private hospital group in the Emirate of Dubai. The Strengthening the Reporting of Observational Studies in Epidemiology (STROBE) statement checklist was used as a framework for reporting this study (von Elm et al., 2008).

## 4 METHODS

Medical records from patients triaged in each of the four emergency centres over a period of six months were evaluated and considered for inclusion. It was expected from the outset and based on interaction with the group's management prior to this study, that the emergency centre patient population would be predominantly low acuity. It seemed reasonable to collect data over a six‐month period for the following reasons: to allow for the collection and inclusion of high acuity cases, the period would represent a similar patient population pattern as that experienced annually, the period included seasonal change that could have an impact on disease profiles, it included a major school holiday and the holy month of Ramadan in the United Arab Emirates. The six‐month period was thus reflective of the population movement in the Emirate of Dubai and inclusive of possible disease dispersion.

Electronically captured data from the four emergency centres were collated by the hospital group medical records department. The data were provided for research purposes in a single Microsoft Excel (2016) spreadsheet. Entries from electronic data captured on each emergency centres information system included the following: number of patients seen (as determined by date stamps), patient demographics (e.g. age, gender and nationality), triage category allocations and principal diagnosis. These entries were anonymized of any patient identifiers (e.g. names, surnames and medical record numbers) prior to the dataset being shared. For each variable described above, the missing data points pertaining to that variable were removed from the sample prior to its analysis. The number of missing data points is reported, and it would have been a result of staff omission and thus obtaining these after the fact would not be possible.

## 5 ANALYSIS

Most of the data were either nominal (e.g. nationalities, gender and principal diagnosis) or ordinal (e.g. triage category allocations) while some were ratio (e.g. age). Distributions were skewed, and non‐parametric descriptive statistics were used with the median as a measure of central tendency. Patient's demographics and principal diagnoses were captured during the patient's journey through the emergency centre and were not affected by the triage system applied. It was known prior to data collection that the four emergency centres used different triage systems; however, this study does not focus on the system used but the outcome of the triage allocations. Although there might have been a predominant triage system advocated by each emergency centre, the actual application by the person conducting triage may have varied. The triage systems used in the four emergency centres were all five‐level systems, as described elsewhere (Dippenaar, 2019). This study quantified the triage category allocations irrespective of the triage system applied.

To determine the variance of triage category allocations between the four emergency centres, an analysis of variance (ANOVA) test was applied. Although the data could be viewed as non‐parametric in nature, preferring the Kruskal–Wallis test, the aim was to determine the variance in total triage allocations (i.e. distribution pattern) between the ECs and not the distribution of actual triage allocations in each EC. The patient demographic pool would thus be similar, and the assumption is that the distribution of triage allocations between the ECs would be similar. A one‐way ANOVA, without replication and a precision level of α < 0.05, was used for the total allocations of each triage category received at each emergency centre. There is a limitation to applying the ANOVA test to the triage categories as they represent levels of acuity and thus have a ranked value: one (i.e. highest acuity) to five (i.e. lowest acuity). The purpose of applying the ANOVA test was to determine whether there was statistically significant variation between the means of the factors and not the variation based on ranked acuity levels. Although two factors existed (e.g. triage category and emergency centre), the limitation of a ranked factor (e.g. triage category) was not suitable for a two‐factor ANOVA analysis. The assumption was that individual triage category allocations varied greatly by nature and thus evaluating the variance of the mean between the individual categories would have provided skewed results. The null hypothesis was therefore that there was no statistically significant difference in the overall distribution of triage category allocations of the four emergency centres.

To determine the correlation of triage category allocations among the four emergency centres, Spearman's correlation coefficient test was applied. A two‐tailed test, to determine relationship in both directions and a precision level of α < 0.05, was used to the total allocations each triage category received at each emergency centre. Spearman's correlation coefficient (non‐parametric) is similar to the Pearson correlation coefficient (parametric) but takes into account the applied rank of the factors (Neideen & Brasel, 2007). The correlation among the four emergency centres regarding their triage category allocations was tested with the triage category as the ranked factor. Interpreting the ANOVA result in combination with a Spearman's correlation coefficient (considering the ranked acuity) provided the best measure of how the triage categories were allocated between the emergency centres.

## 6 ETHICS

The study received ethical approval from the hospital group and the University of Cape Town in South Africa (HREC REF: 744/2014).

## 7 RESULTS

There was a total of 56,984 patient records captured from the four emergency centres. Missing data points identified were as follows: nine (<0.16%) records missing gender, 2001 (3.51%) records missing principal diagnosis and 2,602 (4.57%) records missing triage category allocation. During the six‐month period, the emergency centres near the city centre (Urban) saw the most patients, 48,224 (84.63%) with the southern (Suburban) ones only seeing a small portion, 8,760 (15.37%). There was an overall median of 9,419 (IQR 8510–10483) patients that visited the ECs each month. The number of patients seen between the four emergency centres was of similar proportion each month (IQR: 0.00–0.03).

Of the 56,984 records available with patient nationality data, a total of 173 nationalities were recorded with 42,276 (74.19%) representing the top ten nationalities (Table 1). The largest, single population group was Emirati, from the United Arab Emirates (*N* = 12,361; 21.69%). The Indian population (*N* = 9,158; 16.07%) was the only other nationality that came close to matching the Emirati population.

**Table 1:** Top ten nationalities from patient records (*n* = 56,984)

Rank #	Nationality	*n*	%
1	Emirati	12,361	21.69
2	Indian	9,158	16.07
3	Filipino	3,587	6.29
4	British	3,219	5.65
5	Pakistani	3,117	5.47
6	Jordanian	3,080	5.41
7	Egyptian	2,698	4.73
8	Lebanese	2,529	4.44
9	Syrian	1,311	2.30
10	American	1,216	2.13

**Figure 1:**
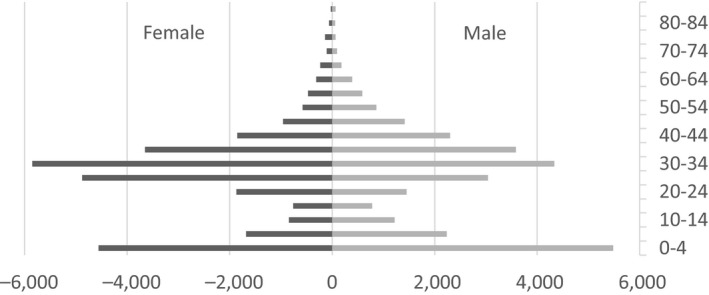
Age and gender distribution from patient records (*n* = 56,975)

Of the 56,975 records available with patient age and gender data, the gender distribution was nearly equal with 28,824 (50.59%) female and 28,151 (49.41%) male records (Figure 1). There were only two age groups that stood out: 0–4 years and 30–34 years with 10,041 (17.62%) and 10,186 (17.88%) records, respectively. The age group considered to represent children (i.e. 0–10 years) consisted of 13,959 (24.50%) records and the age group considered to be the workforce (i.e. 20–50 years) consisted of 35,187 (61.76%) records, together they made up 49,146 (86.26%) of the entire patient demographic. The median age was 29 years (IQR: 10–37).

Of the 54,983 records available with patient principal diagnosis data, it was found that the largest single diagnosis reported during the study period was acute upper respiratory infection (*N* = 7,940; 14.44%) (Table 2). The data reported from the medical records were specific with respect to names of the diagnoses; thus, certain conditions could have been captured under different names in the patient record system. For example, acute pharyngitis (*N* = 2,191; 3.98%), acute nasal pharyngitis (*N* = 879; 1.60%) and acute tonsillitis (*N* = 1,346; 2.45%) could have all been grouped under the name “acute upper respiratory infections,” bringing the total cases to 12,356 (22.47%). The same would apply to abdominal pain, chest pain, headache and fever.

**Table 2:** Top five principal diagnoses from patient records (*n* = 54,983)

No.	Principal Diagnosis	*n*	%
1	Acute upper respiratory infection	7,940	14.44
2	Acute pharyngitis	2,191	3.98
3	Abdominal pain	1,996	3.63
4	Fever	1,801	3.27
5	Infectious gastroenteritis and colitis	1,557	2.83

**Figure 2:**
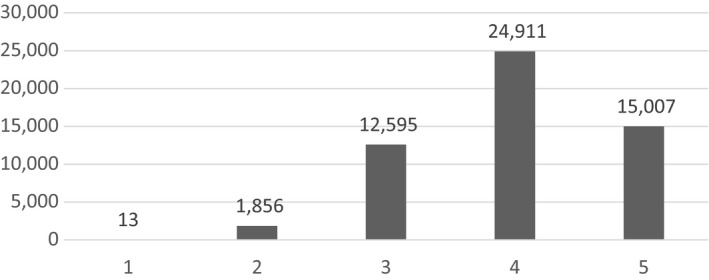
Triage category allocation from patient records (*n* = 54,382)

Of the 54,382 records available with patient triage category allocations data, triage category four was allocated most often (*N* = 24,911; 45.81%). Conversely, category one was only allocated 13 (<0.02%) times (Table 3 and Figure 2). Most of the allocations were made towards the mid to low acuity spectrum (i.e. categories three to five) (*N* = 52,513; 96.56%), whereas high acuity cases (i.e. categories one and two) only made up a small proportion of allocations (*N* = 1869; 3.44%).

**Table 3:** Triage category allocation distribution from patient records (*n* = 54,382)

Category	EC1	EC2	EC3	EC4	Total	%
Total	25,391	20,343	5,149	3,499	54,382	
1	0	13	0	0	13	0.02
2	20	1,833	3	0	1,856	3.41
3	3,909	8,364	278	44	12,595	23.16
4	14,685	6,627	1,986	1,613	24,911	45.81
5	6,777	3,506	2,882	1,842	15,007	27.60

The result of the one‐way ANOVA test (to establish the variation between the ECs): the *F*‐value (*F* = 1.86) was less than the F‐critical (Fcrit = 3.24) with *p* = .18 (*df* = 3). It shows there may be some variation in overall distribution of triage category allocation between emergency centres. Mean correlation among the emergency centres was positive and strong (*r* > 0.85) (Table 4); however, of the four facilities, one of the emergency centres (EC2) did not show the same correlation based on triage category allocations.

**Table 4:** Spearman's correlation^a^ of the triage category allocation distribution

	EC1	EC2	EC3	EC4
EC1	–	0.70	0.90	0.87
EC2	0.70	–	0.60	0.56
EC3	0.90	0.60	–	0.97
EC4	0.87	0.56	0.97	–

^a^ Two‐tailed coefficients with alpha 0.05.

## 8 DISCUSSION

A key finding was that the overall acuity level of the patient population seen at the four emergency centres was low. This was substantiated by the diagnoses profile which indicated traditionally lower acuity principal diagnoses. The amount of high acuity cases overall (i.e. categories one and two) was very small compared with other triage studies (Buschhorn, Strout, Sholl, & Baumann, 2013; Chi & Huang, 2006; Cooke & Jinks, 1999; Eitel, Travers, Rosenau, Gilboy, & Wuerz, 2003; Lee et al., 2011; Vlahaki & Milne, 2009; Worster et al., 2004; Wuerz, Milne, Eitel, Travers, & Gilboy, 2000; van der Wulp, 2010). The way international triage systems are applied is meant to capture the highest acuity patients first and then scale downwards allowing for the safety of over‐triage in the process (Beveridge et al., 1998; Manchester Triage Group, 2006; South African Triage Group, 2012; Gilboy et al., 2012). EC2, the only emergency centre that had notable numbers of high acuity cases, could be contributed to its patient drainage area, situated in the central Dubai Healthcare City (Urban). Incidentally, it was the only one of the four emergency centres that use the Manchester Triage System. National health regulations in the United Arab Emirates at the time prevented private healthcare facilities from seeing major trauma cases, with most to all high acuity cases being seen in state facilities (Dubai Health Authority, 2015; UAE Ministry of Health & Prevention, 2015; Health Authority Abu Dhabi, 2015). Trauma alone contributes substantially to the volumes of high acuity cases in international emergency centres. There are no data or research available to compare acuity levels with other private hospital groups in the United Arab Emirates. Such data would be key in understanding the epidemiological impact of the population and the health care provided.

It would be difficult to directly compare the triage category allocations of the triage systems used against that of other studies done as the settings and circumstances are different. Instead, a comparison was done to evaluate the variance and relationship of the triage category allocations between the four emergency centres. The ANOVA revealed some variation between emergency centre triage allocations, and the null hypothesis could not be rejected, indicating there may not be a statistically significant difference in the distribution of triage category allocations of the four emergency centres. To establish the relationship of triage category allocations among the four emergency centres, Spearman's correlation results showed that three of the four emergency centres correlated well (positively), with similar distribution of their triage category allocations; however, one did not. The specific cause for this aberrance is unknown, as it could have come from the triage system used, the patient population or the operators themselves, which requires further investigation.

Looking at the distribution of patients across the four emergency centres, those near the city centre (Urban) saw considerably more patients than those located towards the south (Suburban) of the emirate. The proportion of patients seen at each emergency centre over the six‐month period was the same, irrespective of the overall volume of patients and suggested a stable and predictable distribution of patients across the four emergency centres each month. This was anticipated since the emergency centres near the city centre was also more readily accessible to communities in the area and converge with transport networks. With a total of 56,984 patients seen at the four emergency centres during the six‐month period, considerably fewer patients were seen when compared with the five public emergency centres in the same region, which was approximately 150,000–160,000 patients (Dubai Health Authority, 2013; Health Authority Abu Dhabi, 2013). This lends the notion that the triage demand on the four emergency centres is relatively low and therefore the triage system used would be under considerably less strain on a day‐to‐day basis.

The overall nationality distribution was a direct reflection of the resident population in the United Arab Emirates (Dubai Health Authority, 2013; Health Authority Abu Dhabi, 2013). The number of Emirati nationals from the United Arab Emirates that visited the four emergency centres is in line with population statistics; however, this was contrary to perceptions in the hospital groups management structures, who believed the native Emirati population made up a significantly smaller proportion of its overall emergency centre patient visits. The patient age distribution indicated a large patient population between the ages of 20 and 50. According to the World Health Organization's annual Statistics report of 2014, this type of distribution is characteristic of both lesser and more developed regions (World Health Organization, 2014). The large workforce group can be attributed to the fact that the largest number of residents in the United Arab Emirates are expatriates that come for work opportunities and are among one of the largest contributors to the United Arab Emirates’ economy (UAE Government, 2015). Local governmental regulations also do not favour residents remaining in the country after retirement and thus most expatriates will leave the United Arab Emirates at an older age (UAE Government, 2015). With this workforce turnover, there may be a skewed and possibly artificially created population distribution. The large female patient population is also contrary to the overall United Arab Emirates population statistics that indicate a male to female ratio of at least three to one (Dubai Health Authority, 2013; Health Authority Abu Dhabi, 2013). A possible reason for this difference considers that most of the male expatriate population are low‐income workforce labourers that do not have access to the private health care. The nationality, age and gender distributions may not have a direct impact on the triage system used, although it may have indirect consequences that may alter the application of a triage system, such as subjective or biased application of triage to favour a specific demographic. With most patients being adults in the working stages of life, the triage system used is not as affected by outlying patient populations, that is children or the elderly. It is not certain what impact these factors would have on a local triage system.

## 9 LIMITATIONS

Extracting information from a database brings with it some limitations as to the authenticity and validity of the data provided. The possibility of missing data points was anticipated but with only three variables showing small proportions of absent data points (gender 0.02%, triage category allocation 4.57% and principal diagnosis 3.51%), it was very unlikely that this undermined the results of this study.

During the analysis of principal diagnoses, it was found that the diagnoses names in the records were very specific. Each entry was thus subjected to the individual physician's preference on which diagnosis name to use. Certain diagnosis names reflected very similar conditions or groupings thereof. For example, acute pharyngitis and acute nasal pharyngitis could have been grouped under acute upper respiratory infection, gastroenteritis could also reflect as abdominal pain, and fever could represent a vast array of conditions. The purpose of the principal diagnosis evaluation was to establish a general overview of the common illnesses treated in the four emergency centres and to determine which illness’ acuities (i.e. high or low) were predominant.

Evaluation of triage category allocations among the four emergency centres was difficult as they used different triage systems to determine patient triage categories. Direct comparisons of the triage categories from differing systems were avoided; however, evaluations among the four emergency centres were possible to measure how the emergency centres applied their triage systems to the patient population. It was thus the distribution of the five triage levels and not their content that was evaluated.

## 10 CONCLUSION

This study has shown that most patients that presented to the four emergency centres of this private healthcare group had low acuity illness profiles and were subsequently allocated lower triage categories. There was an overall low demand on the triage process daily in the four emergency centres; however, this was dependant on their day‐to‐day patient volumes. There were inconsistencies related to the number and distribution of triage category allocations between the four emergency centres and could be related to several factors: the most obvious being the variance in triage systems selection and application. The data and results of this study will help better inform emergency centre policies of this and other private hospital groups in the region in determining the best triage system to suit their patient population needs.

## CONFLICT OF INTEREST

This study was conducted in the emergency centres of Mediclinic Middle East who highlighted triage as a vital component of their business model. The author declares no conflict of interest beyond the support provided by the management of Mediclinic Middle East to undertake this study.
